# Does the immune reaction cause malignant transformation by disrupting cell-to-cell or cell-to-matrix communications?

**DOI:** 10.1186/1742-4682-4-16

**Published:** 2007-05-04

**Authors:** Richmond T Prehn

**Affiliations:** 1 Department of Pathology, University of Washington, 5433 South Hudson St, Seattle WA 98118, USA

## Abstract

**Tumor progression:**

In many (perhaps in all) tumor systems, a malignant cancer is preceded by a benign lesion. Most benign lesions do not transform to malignancy and many regress. The final transformative step to malignancy differs from the preceding steps in, among other things, that it often occurs in the absence of the original carcinogenic stimulus.

**Mechanism of immunostimulation:**

Relatively low titers of specific immune reactants are known to stimulate, but cell-to-cell or cell-to-matrix interactions appear to be major inhibitors of tumor-growth. Therefore, it seems reasonable to hypothesize that the mechanism of immunostimulation may be an interference with cell-to-cell or cell-to-matrix communication by a *sub-lethal *immune-reaction.

**Discussion:**

While the above hypothesis remains unproven, some evidence suggests that immunity may have a major facilitating effect on tumor growth especially at the time of malignant transformation. There is even some evidence suggesting that transformation *in vivo *may seldom occur in the absence of immunostimulation of the premalignant lesion. Positive selection by the immune reaction may be the reason that tumors are immunogenic.

## Tumor progression

It is widely believed that each malignant tumor is a clone of abnormal cells. However, there is great phenotypic and genetic diversity within the cancer-cell population owing to an ongoing process of variation and selection. This continuing process results in "progression"; the term, as used by Foulds [1], describes the progressive changes in the *biological *attributes within a lesion, including dedifferentiation. The term, by definition, is *not *directly related to the tumor's physical growth or extent. I will first review some of the more notable observations that suggest that, during the course of progression, many (all?) malignancies pass through an earlier benign stage before they transform into the malignant state.

Among breeding mice of the C_3_H/An strain, 100% of over 1500 females developed breast cancer [2]. There were numerous HAN (benign hyperplastic alveolar nodules) in each mammary gland as a result of the action of the MTV (milk agent – mammary tumor virus). However, an individual mouse seldom developed more than one carcinoma; the transformation from benign HAN to carcinoma was thus a rare event. The striking feature of the data was that the percentage of surviving mice that developed a breast cancer in each successive month, from the 9th to the 14th, was virtually a constant [2]. The implication is that the benign HAN progressed to one-step-short of malignancy and then awaited a further malignancy-conferring event, an event that, after the 9th month, had a constant probability of occurring in any subsequent monthly interval.

Breast cancer and cervical cancer in women show similar plateaus in the incidence curves, albeit the curves tends to rise slightly with age [2].

Lappé and Prehn studied the development of mouse skin papillomas in response to "initiation" with 3-methylcholanthrene (MCA) [3]. The method of producing the papillomas was to treat the skin of a normal mouse with a sub-carcinogenic dosage of MCA and then graft that skin onto a syngeneic mouse whose immunologic capacity had been raised or lowered by various techniques. The trauma of transplantation served as a "promoter" of the "initiated" skin. A seminal finding was that, in each group, malignant transformation, as a percentage of the papilloma-days at risk, was a constant; *ie*., each papilloma, regardless of its duration and/or presumed immunogenicity, had the same probability of transformation in any subsequent interval of time [3].

Cairns has called attention to a similar observation [4] in the published work of Halpern *et al*.. Halpern reported that the probability, *per *year, of death from human lung cancer stays virtually constant for the next 10–20 years after an individual stops smoking [5]. Apparently, the longer one smokes the larger the number of premalignant lesions that will be induced, but once induced, each lesion retains a constant probability of malignant transformation in each subsequent year.

The preceding five examples suggest that most and perhaps all potential cancers, at some point early in their evolution, consist of benign lesions that very seldom progress to malignancy. Thus, carcinogenesis is not a smooth process of numerous tiny increments, but is usually punctuated by at least one, often prolonged, period of apparent stasis while awaiting, often in vain, for the type of alteration that marks the transformation from a benign lesion into a malignancy [4]. Of perhaps greatest significance is the deduction that the final transformation may be of a nature different from the preceding changes that produced the benign precursor lesion; this is evident from the fact that in most of the five examples cited the *carcinogenic agent was almost certainly long gone at the time of the malignant transformation*. However, in each case the final transforming step occurred with a virtually unchanged probability over successive intervals of time.

## Immunostimulation

Ever since the dawn of the twentieth century, the immune reaction has been associated primarily with the defense against *foreign *invaders, bacterial and viral. This defense involved the killing of the invading organisms and it was readily apparent that such a lethal defense system was absolutely essential for our survival; if it failed, we would be attacked and overwhelmed by the billions of micro organisms that constantly surround us. Nothing seemed to be more obvious than that under no circumstances could an animal tolerate being, in whole or in part, mistaken by its own immune system, for a foreign invader; "horror autotoxicus" in the words of Paul Ehrlich [6].

However, the immune reaction has been found to be exceedingly complex and composed of many constituent parts of which various forms of lymphoid cells seem perhaps of greatest import. It gradually became obvious that autoimmunity could indeed occur and that various autoimmune diseases were all too common [6]; we do not often succumb to such diseases because the immune system usually learns, early in life, what it should and should not attack.

When milder forms of autoimmunity occur, the target cells may not be killed, but may instead undergo an extensive hyperplasia. To the pathologist, this association is as common as "bread and butter". That the immune reaction is actually the cause of the hyperplasia is most easily illustrated by the analysis of a mild alloimmunity in which it was possible to titrate the strength of the reaction. Chai tried to develop inbred rabbits [7]. Therefore, numerous generations of brother-sister matings were enabled. As the animals became more inbred, reciprocal skin grafts were performed to test the genetic identity that was gradually approached. As the animals neared homozygosity, the skin grafts were no longer rejected as is the case with foreign grafts. Instead, they underwent a *chronic *acanthosis and epithelial hyperplasia. Of course, animals that are *fully *inbred accept isografts with only a minor and temporary inflammatory reaction. Numerous other examples, in which an immune reaction appears to have produced hyperplasia in normal tissues, have been documented [8]. If we admit that an immune reaction can produce hyperplasia in an overtly normal target tissue, can one surround a tumor with immune lymphoid cells and insist that they are inhibiting tumor growth?

**Figure 1:**
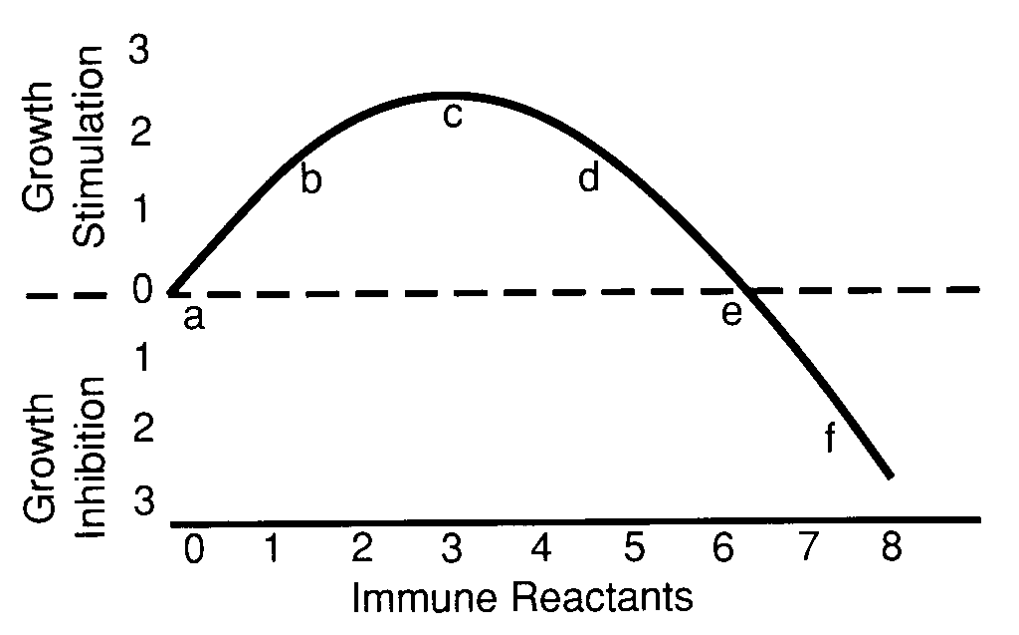
The immune response curve (IRC), idealized from data in [10]. The lettered and numbered points are inserted only to aid the discussion.

The fact that an immune reaction may, under some circumstances, act to enhance rather than inhibit neoplastic growth has been known for many years [9]. The first convincing demonstration that more might be involved than a mere blockage of a defensive aspect of immunity was probably a study with 3-methylcholanthrene (MCA)-induced mouse sarcomas in a totally syngeneic system [10]. A fixed number of sarcoma cells was mixed with varied numbers of immune spleen cells, *ie*., spleen cells from mice that had previously grown the same tumor. The resulting tumor growths, when such an admixture was implanted subcutaneously in radiated and thymectomized hosts, were compared with controls consisting of that same tumor mixed with nonimmune spleen cells or with no spleen cells. It was found that relatively *small numbers of admixed immune cells stimulated tumor growth while larger numbers of the same cells were inhibitory *(figure 1 – the immune-reaction-curve or IRC). The fact that the host animals had been radiated and thymectomized suggested that a blockage of host immunity was an unlikely explanation for the apparently stimulated growth. In subsequent years, a large literature has appeared suggesting that the immunostimulation phenomenon may be exhibited by a variety of tumor systems and by a variety of immune reactants, such as antiserum, macrophages, NK cells, cytotoxic lymphoid cells, *etc*. acting either separately or in algebraic aggregate [11-13]. At this time, I tentatively venture the hypothesis that any entity capable of binding an antigen on a neoplastic cell can probably exhibit the same phenomenon; *ie*., stimulation of the target cells at low titer, but tumor-inhibition or killing at high.

While this discussion has emphasized the role of the shear *quantity *of immune reactants, the immune response has many component parts and is very complicated; the quality of the reactants at the antigenic site is also influential [14]. (See Outzen's review of older literature [13]).

Analysis of the effect of immunodepression of the recipient mice, in the previously discussed Lappé/Prehn papilloma study, showed that a moderate immunodepression increased papilloma-incidence, papilloma-progression to malignancy, and delayed papilloma-regression [3], a result consistent with either increased immunostimulation or decreased immunoinhibition of the papillomas depending upon whether the reaction's location was to the left or to the right respectfully of point "e" on the immune -reaction-curve [15] (Figure 1); in either case, the reaction would be to the right of "c". The important point for the immediate discussion is that the immune reaction definitely influenced the growth of both premalignant and malignant lesions.

By contrast, the incidence of *rectal *carcinoma has been reported to be dramatically *less *in immunodepressed kidney-transplant patients [16]. Such a phenomenon, if caused by the immunosuppression, would place the lesion to the left of "c" on the IRC (figure 1). This is the only region on the IRC where a reduced immune-reaction would result in less tumor growth. However, there is overwhelming evidence, from surgical specimens of malignant *colorectal*-lesions, that a heavier immune-cell infiltrate is associated with an improved prognosis [17]. This observation would place the reaction somewhere on the slope to the right of "c". These two somewhat different results can be reconciled since it is apparently only the *rectal *carcinomas whose incidence is decreased by immunodepression; when colonic lesions are considered together with the rectal lesions, the average positon on the IRC is shifted, as would be expected, to the right.

## Immunostimulation of malignant transformation

As shown with the MCA-induced skin papillomas [3], immunity probably acts throughout the life of a tumor and is not limited to the period of malignant transformation. However, a reinvestigation of the skin-papilloma system, with a slightly modified protocol, gave added insight [18]. Andrews' methodology was to expose mouse skin to the MCA before transplantation to an *allogeneic *rather than to a syngeneic host. In order to permit the grafts to survive in the allogeneic host, the host animals were maximally immunodepressed by thymectomy, x-radiation and weekly injections of antithymocyte serum. No evidence of surviving immune function could be found, so the reaction was presumably near "a" on the IRC. Nonetheless, about 80% of the induced papillomas regressed and, most surprisingly, *none progressed to malignancy*. Apparently an immune reaction was necessary, in this system, for malignant transformation (See discussion in [12]).

The Andrew's experiment was unique in its extreme degree of immunodepression [18]. An abundance of studies in mice with varied immune capacities show that the degree of immunodepression is critical for carcinogenesis and tumor growth. However, the titrations were not carried to the extreme used by Andrews, but covered the range in which an intermediate level of immune capacity was associated with maximal tumor growth while either less or more capacity produced relatively less growth [19]. In experiments patterned upon the methodology used by Lappé and Prehn, skin tumor production in germ-free nude mice was consistantly less than in immunologically reconstituted nudes [13]. However, implants of highly immunogenic, and *only of highly immunogenic*, tumors grew better in irradiated than in non-irradiated nudes [20]. These observations suggest that the reactions to the tumors in the nudes fell somewhere to the left of "c" but, in contrast to the work of Andrews, well to the right of "a" on the IRC (Figure 1).

The transformation of benign nevi into melanomas also suggests a critical role for immunity. Most cutaneous malignant-melanomas probably arise in preexisting benign-nevi (albeit many of the nevi may be too small to be grossly detected so this relationship is not secure). The incidence of benign-nevi is increased dramatically in people who are immunodepressed to make kidney-allografts possible [21]. Nevi are also more numerous in patients suffering from AIDS which suggests that it is indeed immunosuppression *per se *that is responsible for the increase [21]. I argue that it is probably not lack of immunosurveillance that accounts for the increased incidence of nevi in immunodepressed patients; there is little or no immune-cell infiltrate associated with nevi in immunonormal individuals. It seems more likely that immunodepression weakens an already weak immunity and, by so doing, moves the reaction along the curve in figure 1 from near "d" or "e" toward "c", thus increasing the level of tumor-stimulation and so increasing the number and size of nevi. While I feel it to be unlikely, a not mutually-exclusive explanation cannot be dismissed; namely, that since melanocytes can serve as antigen presenting cells [22], the excess of nevi in immunodepressed patients might be the result of a compensatory hyperplasia.

Although an immune reaction may play a stimulatory role in the life of a nevus, at the time of transformation of a nevus into a malignant melanoma, the immune response is markedly increased as judged by the often heavy immune-cell infiltrate. Whether it increases sufficiently to actually become inhibitory is not clear; it has been claimed that the heavier the infiltrate in the mature lesion, the better the prognosis [23]. This observation, if substantiated, would be consistent with the heavier infiltrate being *either *less stimulatory or being more inhibitory (it would be somewhere on the slope to the right of "c" on the IRC).

The significant point for the immediate discussion is that the heavier immune-cell infiltrate, in conjunction with the transformation of a nevus into a malignant melanoma, suggests an amplified role for immunity *during the process of transformation per se*.

## Societal control of malignancy

The well-established fact that cancer is often held in check by interaction with matrix or with neighboring cells has been well reviewed, most recently by Bissell *et al *[24] and by Rubin [25]. In fact, the cancer phenotype can sometimes be normalized *in vivo *by forced contact of the cancer cells with surrounding parental normal cells. Suitable markers, as well as recovery of the original cancer phenotype, testify to the fact that a phenotypic change from cancer to normal and back again did actually occur in a tumor-cell lineage [25,26].

Interference with gap-junctional-communication may play a part in the societal control of tumor growth [27], but is apparently not always necessary [25]. In fact, in the case of the hyperplastic nodules of the mouse breast, a nodule implanted into a gland-containing fat-pad is inhibited by the presence of the normal glandular-tissue without the necessity of direct contact [28]. By contrast, an implant of overt carcinoma is not inhibited by the presence of normal gland [29]. Thus, as might be expected, it seems that there are probably a variety of mechanisms by which the normal cellular society tends to keep deviant cells under control, but these controls lose effect as the deviants become progressively less normal and as the societal controls diminish with the aging of the individual [25].

## Mechanism of immunostimulation

Inasmuch as immunity can be a tumor-stimulator, but cell-to-cell or cell-to-matrix interactions appear to be major inhibitors [24-26], it seems reasonable to suggest that the mechanism of immunostimulation may be an interference with cell-to-cell or cell-to-matrix communication by a *sub-lethal *immune-reaction. Immunity is dependent upon interactions with cell-surface antigens and so would appear to be a logical mechanism for interfering with such communications. However, direct evidence in support of this hypothesis is not presently available.

## Discussion

The hypothesis that immunostimulation of tumor growth may result from interference with cell-to-cell communications seems likely, but remains unproven; the idea that immunostimulation is peculiarly effective during transformation from benign to malignant is even more tenuous. Melanoma is the only tumor system, of which I am aware, in which there is seemingly clear evidence of a profound change in the immune infiltrate *at the time of transformation to malignancy*. The phenomenon may be universal, but evidence from other systems does not appear, at present, to be available.

However, a role for immunity in malignant transformation *per se *is also suggested by the failure, already discussed, of mouse skin-papillomas to undergo transformation if the host's immune-capacity is not only diminished, but virtually abolished [18] (see discussion in [12]). This observation suggests, quite independently of observations of immune-cell-infiltrations, that immunity may specifically facilitate rather than inhibit malignant-transformation and may even be necessary for such transformation *in vivo*. At present this idea remains an attractive hypothesis. If it were correct, one would be faced with envisioning some plausible mechanism for the postulated increased blockage of intercellular communication at the time of transformation.

The final step in the transformation process might be a new mutation or some epigenetic alteration that, in the context of the benign lesion, would occur with an unchanging probability over time and that would increase the effective immunogenicity of the tumor.

Cairn's "immortal strand" might, if that thesis were correct, provide one possible explanation [4]. He proposes that the final step in transformation may occur when the postulated immortal chromatid strand, preserved in a stem-cell, is effectively replaced when the stem cell itself is replaced by a newly dedifferentiated daughter-cell; the daughter cell is postulated to be more capable of symmetrical division, *ie*., the old stem-cell is superseded by a new defective-stem-cell that is less subject to the societal inhibitions that usually prevent symmetrical division of stem-cells [4]. The new, replacement stem-cell might carry accumulated antigenic mutations not present in the superseded immortal strand and thus provide, concurrently with malignant transformation, greater antigenic stimulation; this in turn might provide increased stimulation of tumor growth if the reaction were to the left of "c" on the IRC (Figure 1).

If the immune reaction is indeed a necessary adjunct to malignant transformation, the lower incidence of some cancers, such as rectal [16] and breast [30], in immunodepressed patients is probably explained by the movement of the reaction further to the left of "c" on the IRC. However, the incidences of most other tumors are probably mildly elevated in immunodepressed patients [16] and some, such as skin cancers and lymphoreticular tumors, markedly so. This seeming paradox is easily explained by the fact that various types of tumors carry differing immunogenicities; the MCA-induced skin papillomas of the mouse are relatively immunogenic and their incidence is increased by moderate immunodepression. If a tumor's immunogenicity places it to the right of "c" on the IRC, immunodepression will result in a higher incidence; to the left of "c", immunodepression will result in a lower incidence. In general, most viral or chemically induced tumors will be more immunogenic and tend to fall to the right while so-called spontaneous tumors will tend to fall to the left of "c". Unfortunately, the immunogenicities of most human tumors can presently be judged only by the effect of immunodepression on their incidences.

If the above argument is correct, the bottom line would appear to be that tumors that fall to the left of "c", such as rectal carcinomas and mammary carcinomas will probably be relatively difficult to treat by immune enhancing measures such as vaccines (these tumors might actually be stimulated by an effective vaccine), while skin tumors and lymphomas, being usually to the right of "c", may be better candidates for vaccine therapies. Furthermore, in keeping with the ideas I have been forwarding, it may not be too big a stretch to suggest that the reason tumors have antigens is so that they can become tumors; without antigens and the resulting immunostimulation, they might never begin to grow!

## Abbreviations

IRC = immune response curve; MCA = 3-methylcholanthrene

## Competing interests

The author(s) declare that they have no competing interests.
